# Fat Embolism Syndrome With Cerebral Involvement: An Underrecognized Complication of Long Bone Fractures

**DOI:** 10.7759/cureus.22816

**Published:** 2022-03-03

**Authors:** Quang L Nguyen, Benadin Varajic, Samuel B Reynolds, Karim El-Kersh

**Affiliations:** 1 Division of Pulmonary, Critical Care, & Sleep Disorders Medicine, University of Louisville School of Medicine, Louisville, USA; 2 Department of Internal Medicine, University of Louisville School of Medicine, Louisville, USA; 3 Division of Pulmonary, Critical Care, and Sleep Disorders Medicine, University of Louisville School of Medicine, Louisville, USA; 4 Division of Hematology and Oncology, University of Michigan, Ann Arbor, USA; 5 Pulmonary and Critical Care, University of Nebraska Medical Center, Omaha, USA

**Keywords:** traumatic long-bone injuries, encephalopathy, respiratory failure, cerebral fat emboli, fat embolism syndrome

## Abstract

Fat embolism syndrome (FES) occurs when fat particles are aberrantly distributed into the microcirculation, and it often manifests as either hypoxemia, neurological deficit, or petechial rash. Although cases have been reported in the literature since the twentieth century, no formal diagnostic criteria have been universally adopted, and FES remains a diagnostic challenge. We present a unique case of FES from a long bone fracture, leading to pulmonary embolism with paradoxical arterial embolization and cerebral infarction, and provide a review of the related literature.

## Introduction

The incidence of subclinical fat emboli has been described in more than two-thirds of orthopedic trauma cases in a review by Bulger et al., with an even higher incidence when serum samples are drawn in close proximity to the trauma site [1-2]. If there are numerous fat particles (i.e., >100 particles per mm²), the clinical phenomenon of FES often results and is itself classically characterized by a triad of respiratory distress, petechial hemorrhage, and encephalopathy. This constellation of symptoms is thought to be secondary to direct tissue ischemia from mechanical obstruction of blood vessels and subsequent activation of an intracellular inflammatory signaling cascade [3]. Neurologic manifestations are non-specific, perhaps due to the intrinsic variability in the caliber and tortuosity of intracranial arteries, and range from personality changes to stupor and even coma depending on the affected territory. The exact incidence of FES stemming from traumatic long bone injuries varies in the literature, from as low as 0.9% when clinical criteria are used for diagnosis to as high as 29% in postmortem autopsies [4]. Cerebral fat embolism (CFE) has been described in about 10% of FES cases based on clinical criteria, but the true incidence is also unclear due to an absence of definitive diagnostic testing [5]. When clinical criteria are combined with neuroimaging, the reported incidence of CFE from long bone injuries is 0.9%-2.2% [3-6].

## Case presentation

An 83-year-old female presented to the hospital with generalized unresponsiveness after a mechanical fall at home. Past medical history was significant for coronary artery disease and transcatheter aortic valve replacement for severe aortic stenosis one year prior, which itself was complicated by an embolic left hemispheric stroke requiring dual antiplatelet therapy. After admission, the patient became increasingly hypoxic and hypotensive, prompting intubation, mechanical ventilation, and vasopressor support.

Plain radiography on admission revealed right femoral neck and right clavicular fractures. Initial brain magnetic resonance imaging (MRI) demonstrated no acute findings, and lower extremity venous Doppler was negative for deep vein thrombosis. Chest radiograph, however, demonstrated bilateral infiltrates, and subsequent transthoracic echocardiogram (TTE) provided visualization of moderate right ventricular enlargement and moderate global hypokinesis with septal flattening in systole, consistent with right ventricular pressure overload. The left ventricular ejection fraction was normal (Video 1).

**Video 1:** Transthoracic echocardiogram Transthoracic echocardiogram (TTE) with subcostal four-chamber view demonstrating left ventricular septal flattening. The right ventricle is enlarged with free wall hypokinesis and dynamic invagination of the apical wall.

**Figure 1:**
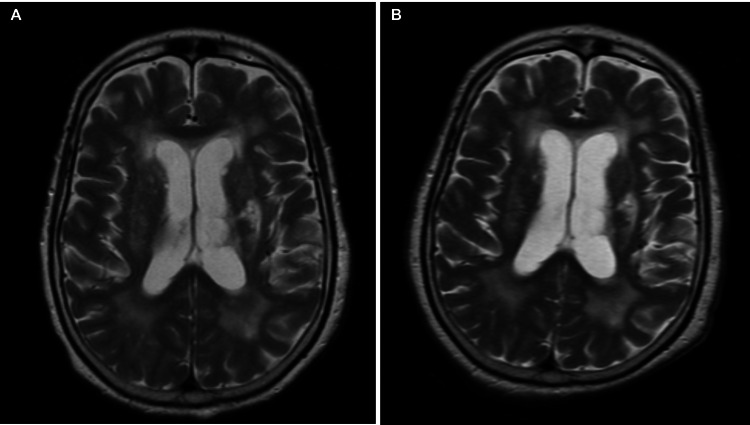
MRI brain without contrast MRI showed numerous punctate focal lesions on T2 hyperintensity involving the gray-white matter junction of bilateral cerebral hemispheres and within the left middle cerebellar peduncle (A), which were new compared to admission MRI (B).

A continuous heparin infusion was initiated empirically; chest computed tomography (CT) with contrast was deferred due to poor renal function and its low likelihood to alter clinical decision-making. Due to a poor neurologic response off sedation, a second brain MRI was done on day three of hospitalization, which demonstrated new punctate focal lesions on T2 hyperintensity involving the gray-white matter junction of both cerebral hemispheres and within the left middle cerebellar peduncle (Figures 1A-1B).

**Figure 2:**
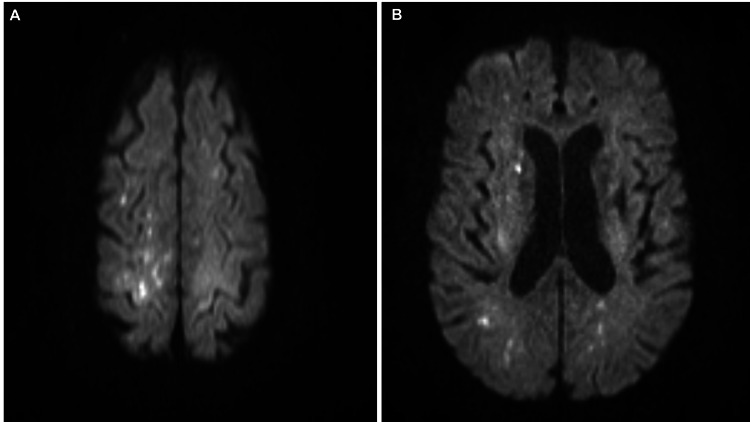
MRI brain DWI sequencing Diffusion-weighted imaging (DWI) sequence showing scattered punctate microhemorrhages seen at the gray-white matter junction of bilateral frontal and parietal lobes

Additionally, the diffusion-weighted imaging (DWI) sequence revealed new, scattered punctate microhemorrhages at the gray-white matter junction of the frontal and parietal lobes bilaterally (Figures 2A-2B).

A repeat TTE, instead of transesophageal echocardiogram (TEE) due to thrombocytopenia, was performed on day four of hospitalization. The repeat TTE showed no valvular vegetation and complete normalization of the right ventricular findings noted on the prior study; shunting was not appreciated on an agitated saline bubble study. The patient remained off sedation with poor neurologic responsiveness that included obtundation in addition to the absence of any purposeful movements; pupillary, corneal, oculo-cephalic, and gag reflexes were still present. The patient was eventually transitioned to comfort care.

Although the differential for encephalopathy is broad, the star-field distribution pattern of lesions on MRI narrows the diagnoses to diffuse axonal brain injury, cardiogenic cerebral emboli, cerebral vasculitis, or hemorrhagic intracranial metastases. In the presented case, the patient was diagnosed with cerebral FES due to her clinical picture in the context of an MRI that demonstrated innumerable scattered punctate foci of faint restricted diffusion in the gray-white matter of bilateral cerebral hemispheres. In the absence of cutaneous changes on physical examination, she did meet two major criteria (respiratory distress/hypoxemia with a partial pressure of oxygen (PaO2) of <60 mmHg and cerebral symptoms in non-traumatic brain injury) of Gurd and Wilson’s criteria for fat emboli syndrome (Table 1) [1].

**Table 1:** Gurd and Wilson’s criteria for fat emboli syndrome Source: [1] ESR: end-stage renal disease

Requires two major criteria or one major and four minor criteria for diagnosis
Major Criteria
Respiratory distress or hypoxemia with PaO₂ <60 mmHg
Petechial rash or cutaneous changes
Cerebral symptoms in non-traumatic brain injury
Minor Criteria
Tachycardia (≥110 bpm)
Fever (≥101.3° F)
Jaundice
Renal injury
Retinal injury
Acute anemia
Acute thrombocytopenia
Elevated ESR
Fat macroglobulinemia

We hypothesize that the fat emboli likely resulted in acute hypoxic respiratory failure and initial right ventricular overload with subsequent paradoxical embolization through pulmonary microcirculation, resulting in cerebral fat deposits and normalization of right ventricular pressure.

## Discussion

The presentation of FES is variable and depends on the organ system whose microvasculature has been impacted by fat emboli. Damage to the retina, kidney, liver, and heart has been reported, although the triad of petechial hemorrhages, respiratory distress, and altered mental status is the most common clinical presentation [1-2]. Cutaneous manifestations take on the form of a petechial vest-like structure in the axillary or trunk region and occur in up to 50-60% of cases but can be transient [1,7-8]. Pulmonary complications occur in up to 75% of cases, and 39% of patients develop severe hypoxemic respiratory failure that can be indistinguishable from acute respiratory distress syndrome [1,7]. Neurologic symptoms are the most common and occur in 85% of cases [1,7]. CFE is a sequela of FES and is diagnosed when neurological involvement is either seen on imaging or is the main symptomatic manifestation of the disease [3,6]. The condition occurs when fat emboli are scattered into the cerebral microvasculature with disruption of the blood-brain barrier and subsequent cerebral ischemia [8]. Associated pulmonary distress or petechia may be present with the cerebral involvement but is not necessary to make the diagnosis.

Since the first reported incidence of fat embolism over 150 years ago, several theories have been proposed for the mechanism by which fat emboli are distributed into the arterial circulation [1]. The mechanical theory postulates that traumatic long bone injuries lead to the exposure of bone marrow contents and a subsequent increase in intramedullary pressure, which induces massive extravasation of fatty bone marrow into the open venous sinusoid [1,9]. This allows the fat droplets to freely travel through the venous system at high velocities to dislodge in the lungs, where they can interact with the lung parenchyma [9-10]. Local damage to and turnover of lung tissue then causes an increase in both intrathoracic pressure and retrograde right-sided intracardiac pressures, which force the emboli to pass through a patent foramen ovale (PFO) into the left atrium and ultimately the arterial circulation, i.e., paradoxical embolism. One study even demonstrated an incidence of fat embolization in 41% of patients through intraoperative transesophageal echocardiography (TEE) during fixation of long bone fractures [1]. Fat emboli can also directly pass from the venous to arterial circuits through lung microvascular capillaries and through intrapulmonary physiologic shunting [9-10]. This phenomenon, however, is only seen when fat particles are of a certain number (greater than 100 particles/mm²) and size (7-10 micrometers) [3]. It is believed that larger particles (i.e., those larger than 20 micrometers) are blocked by the luminal barrier between pulmonary microvascular endothelial cells [3].

The biochemical theory points to a local inflammatory response at the site(s) of fat emboli deposition, which itself is characterized by a surge in catecholamines as a stress response to microtrauma to the affected capillary bed. This surge then induces a liberation of free fatty acids, which interact with lung lipases, leading to the activation of the intrinsic apoptosis cascade and subsequent damage to both lung parenchyma and capillary endothelium. The specific disruption of capillary membranes is thought to be secondary to a loss of endothelial cells, which causes an increase in tissue permeability; inflammatory-mediated damage to surrounding tissues then results in interstitial hemorrhages and edema [1,8-10].

And although these two theories differ in mechanism, both pathways are likely contributory to the underlying injury FES. The mechanical phase causes blockage of capillaries and resultant veno-arterial shunting; the subsequent biochemical phase induces inflammation and apoptosis that causes damage to surrounding tissue [8]. Moreover, activation of the apoptotic cascade can also disrupt systemic circulation, leading to poor tissue perfusion and ultimately multiorgan damage [11].

Making the diagnosis of FES can be challenging, and although Gurd and Wilson’s criteria (Table 1) have been widely adopted in clinical practice, no formal diagnostic criteria have been validated with large cohort studies [1]. As for neuroimaging, MRI is the most sensitive diagnostic modality, where changes can be seen as early as four hours after the onset of neurologic symptoms [2-3]. MRI is superior to head CT due to increased tissue resolution and gray-white matter differentiation. A case series published by Gupta et al. demonstrated three patients with traumatic long bone injuries who had both respiratory failure and encephalopathy in which CT head was unremarkable, but early MRI demonstrated evidence of CFE [2]. Early imaging is also critical, as CFE is typically seen between 24 and 72 hours of fracture; one retrospective study reported an average presentation of 18.4 hours after the initial insult [4,9]. It is also worth noting that the findings on MRI represent ischemic occlusions of cerebral arterioles, which causes cytotoxic edema on DWI in the acute stage and vasogenic edema on T2-weighted sequences in the later stages [2-3,6]. Therefore, although CFE may be confounded by other neurological processes, such as stroke or anoxic brain injury, early MRI in patients with neurological symptoms following fracture in the absence of other causes can hasten the diagnosis and allow for appropriately targeted management.

The clinical presentation and ultimate prognosis of CFE are variable and depend on the disease burden. Patients with acute respiratory distress syndrome generally have the worst prognosis, as most deaths are attributed to respiratory failure [1,7]. This was evident in the presented case, as the patient’s worsening respiratory failure contributed to mortality despite an early diagnosis of CFE. The severity of neurologic symptoms also depends on the distribution and size of ischemia, although the subsequent resolution of edema in less than two months corresponds to a favorable outcome, as the effects of CFE are often reversible. Worse outcomes are seen in patients with persistent and/or multiple infarctions two months after the insult [11]. Takahashi et al. correlated signal abnormalities on MRI (graded on size and distribution) with specific clinical findings that included the onset of CFE, degree of neurological impairment, and Glasgow Coma Scale scoring (Table 2) [2,11].

**Table 2:** Takahashi grading of brain lesions on MRI T2 images Source: [2]

Gradings	MRI Findings
Grade 0	No abnormality
Grade 1	Several small spotty high-intensity lesions seen in deep white matter or deep brain structures
Grade 2	Either many small spotty high-intensity lesions or macular lesions that represented confluent spotty lesions in the deep white matter or deep brain structures
Grade 3	Large macular high-intensity lesions in the deep white matter

Despite CFE traditionally being associated with poor neurologic outcomes, several reported cases have described full neurologic recovery with only minor residual deficits through prompt diagnosis and symptomatic care. The majority of patients who receive appropriate supportive care, in fact, can recover from both neurologic and pulmonary sequela, with an overall mortality of only 7-10% [1,4]. In one case series by Gupta and colleagues, four patients who had sustained traumatic long bone injuries were reported to have developed respiratory failure and encephalopathy with MRI findings characteristic of CFE. Following early operative fixation of fractures, however, 75% had a near-complete regression of lesions on repeat MRI [2].

Treatment in patients diagnosed with CFE begins with prompt diagnosis and general supportive measures. Proposed disease-directed therapies have been trialed but have largely been unsuccessful to date. Attempts to decrease free fatty acid mobilization and lipolysis have not shown any clinical benefit, and anticoagulation may increase the risk of bleeding [1,3]. A meta-analysis of seven randomized clinical trials utilizing prophylactic high dose corticosteroids in patients with long bone fractures, however, demonstrated a risk reduction of 77% [1]. The theorized biologic mechanism is that corticosteroids stabilize the phospholipid bilayer comprising cell members, thereby limiting free fatty acid levels and inhibiting complement-mediated leukocyte aggregation [1]. Despite this, the use of steroids is controversial, and further examination of the same trial showed no difference in mortality compared to control patients receiving standard prevention and supportive treatment [1,3,6,11]. Still, based on this study, some clinicians utilize methylprednisolone 6-90 mg/kg as FES prophylaxis in patients with long bone fractures [1]. Regarding novel therapies, a more recent study by Xiong et al. proposed that urinary trypsin inhibitor (UTI), a Kunitz-type protease, could be used to attenuate the injury to the blood-brain barrier following fat embolization by blocking the activation of pro-inflammatory and apoptotic pathways in endothelial cells to prevent vasogenic edema [8]. This study, however, was only conducted in vivo and has not yet been expanded to humans. Therefore, the cornerstone of treatment remains prevention through early operative fixation and timely, supportive care of the affected organ systems as necessary. In the setting of severe neurological impairment, supportive care should include both airway protection and minimization of lung injury with intubation and mechanical ventilation for respiratory support, if needed.

## Conclusions

FES with CFE, in summary, are potentially fatal sequelae of long bone fractures, and although there are correlative examination and imaging findings, the diagnosis remains clinical. For patients who experience traumatic long bone fractures and/or injury and then develop respiratory failure with encephalopathy, a diagnosis of CFE should be considered. If clinical suspicion remains high, an MRI of the brain with both diffusion and T2-weighted imaging should be obtained. And while disease-directed therapies are under development, the optimal management remains preventive and may be achieved through early operative fixation of long bone fractures and correction of hypoxemia to maintain end-organ and tissue perfusion.
